# Editorial: Intersection of diet, intestinal microbiota, and their metabolites on cancer prevention

**DOI:** 10.3389/fnut.2023.1358428

**Published:** 2024-02-01

**Authors:** Nancy D. Turner, Tiffany L. Weir, Jacques Izard

**Affiliations:** ^1^Department of Food Science and Human Nutrition, Michigan State University, East Lansing, MI, United States; ^2^Department of Food Science and Human Nutrition, Colorado State University, Fort Collins, CO, United States; ^3^Department of Internal Medicine, University of Nebraska Medical Center, Omaha, NE, United States; ^4^Frederick F. Paustian Inflammatory Bowel Disease Center, University of Nebraska Medical Center, Omaha, NE, United States; ^5^Fred and Pamela Buffett Cancer Center, University of Nebraska Medical Center, Omaha, NE, United States

**Keywords:** nutrition, microbiome, postbiotics, metabolome, dysbiosis

As lifespan and cancer incidence continue increasing worldwide, developing preventative interventions that integrate the positive attributes of beneficial changes in lifestyle and diet, resulting in exposures to health-promoting small molecules and microbiota carries tremendous appeal and challenges. Accumulating evidence suggests these factors interact via complex mechanisms that can potentially suppress or aggravate pathways underlying a diverse range of diseases. The intersection of nutrition with microbiology and immunology has emerged as an active research continuum spanning molecular techniques to clinical interventions. Recent studies provide both conceptual and empirical insights that leverage bioactive dietary constituents, acting on or through the microbiota, against conditions involving inflammatory processes. These findings have been obtained in a wide range of scientific models, leading to a better understanding of association and/or causality.

A compelling line of investigation is the use of whole foods for mitigation of cancer risk or recurrence. Although conceptually simple, it is challenging to decipher mechanisms associated with the intake of a complex food containing multiple phytochemicals and bioactive components. The drive to understand the underlying mechanisms involved in beneficial foods or diets has forced the scientific community to use the reductionist approach of studying purified phytochemicals such as inositol hexaphosphate (IP6), found in grains and legumes. While the human digestive system cannot metabolize these phytates, the gut microbiota has been shown to metabolize or dephosphorylate them producing compounds that can impact both the microbiota and the intestinal epithelium. Lan et al. (1) used a murine animal model to investigate IP6 impacts on the microbiome, host gene expression, and CRC metastasis. In their study, IP6 altered gut microbes, specifically increasing Lactobacilli and reducing *Escherichia* as well as reducing metastasis-linked gene expression in the mice. Although the mechanisms remain unclear, increased *Lactobacillus helveticus* was correlated with a suppression of *Tnfrsf1b* gene expression, and the modulated microbial profile was associated with beneficial immune responses. Anti-cancer effects were evidenced by reduced liver metastasis, cecal tumor weights, and modulated NF-kB activity alongside microbiota changes. However, details on the specificity of IP6's actions via certain microbes or cytokines impact on metastatic pathways, and whether clinical translation is warranted remain to be determined.

The use of dietary supplements is well accepted, provides a more standardized approach, and can be less daunting than dietary changes. The integration of a neurocognitive behavioral aspect to the nutrition-inflammation-microbiota axis challenges us to adopt a more integrative understanding of body systems. Tuska et al. (2) invite us to explore supplementing folic acid and protein yielding intriguing results in a murine model when introducing exercise. Cognitive decline was apparent in protein/folic acid deficiency as assessed by nest-building behavior. The consumption of a high protein diet resulted in the most distinct fecal microbiota composition effects with a decrease of the Firmicutes/Bacteroidetes ratio, while groups including exercise observed the opposite trend. Folic acid and protein supplementation combined with exercise decreased liver IL-6 and NF-kβp65, and increased liver CASP3 and muscle TNF-α. Varying the dietary intake of nutrients, folic acid and protein, from deficiency to excess alters the microbiome, and inflammatory markers in different organs in the context of exercise. All these parameters have been shown to be modulators of both cancer risk and progression as well as neurocognition. Untangling the opposing effects of exercise vs. macronutrient excess will advance progress toward precision nutrition in cancer prevention and treatment.

Postbiotics offer emerging prophylactic and therapeutic alternatives/complements to prebiotics and probiotics by utilizing bioactive metabolites or other components (i.e., cell wall, proteins, etc.) of beneficial gut microbes. In some cases, like in cancer patients, this emerging approach could improve standardization and safety compared to administration of live microorganisms. Song et al. (3) discuss how microbial components and derived molecules could be deployed in the context of prevention and activity against cancer with a focus on colon cancer. Short-chain fatty acids, structural components like exopolysaccharides, host molecules modified by microbial activity, such as secondary bile acids, and bacterial enzymes demonstrate anti-cancer activities. The mechanisms vary greatly from one component to another. The mode of action encompasses inhibition of cancer cell proliferation, induction of apoptosis by interfering with characteristic signaling pathways that become dysregulated during malignant transformation, modulation of cancer-associated gene expression, immunomodulatory activity, and restoration of commensalism.

Host signaling, influenced by diet and microbiome modification, offers a novel and accessible path to support pharmaceutical treatment. Hilakivi-Clarke et al. (4) make the case for estrogen receptor alpha positive (ERα+) breast cancers where modulation of estrogen receptors by inhibition (for ERα) or activation (ERß) could lead to a positive impact on cancer immunotherapy effectiveness. The gut microbiota, particularly through generation of high fecal short-chain fatty acid levels, strongly influence whether immune checkpoint blocker (ICB) treatment will be effective. ERα signaling can hamper anti-tumor immunity, so inhibiting this pathway may improve immunotherapy outcomes. Foods and dietary components like fiber and phytochemicals, or ketogenic diets beneficially alter gut microbiota and its downstream immunomodulatory effects, and can decrease circulating estrogen levels, which may potentiate ICB therapy. Specific phytochemicals in plant foods can preferentially bind ERß and G-protein coupled estrogen receptor, which in turn may enhance treatment options. Clinical trials are in progress to determine the effectiveness of dietary intervention as a supportive component of pharmaceutical treatment. This approach opens opportunities to modify the threshold of so-called refractory disease.

These multiple lines of evidence make a case that precision medicine and/or wellness necessarily require transdisciplinarity. We need to analyze, synthesize, and harmonize information/data between disciplines into a coordinated and coherent whole. Translating this work into clinical practice or modifying individual habits requires us to integrate social and health sciences in a humanities context. This requires the inclusion of stakeholders in problem-solving approaches that are applied to tangible problems. The challenge remains on how to integrate data from opposing effects. Advances in this area will assist in customizing the optimum levels of drug, nutrient, pre-/pro-/post-biotics, physical activity, and other possible interventions, including behavioral modifications. Inclusive research initiatives will provide us with opportunities to refine disease prevention/treatment approaches to enhance their effectiveness in individuals.

## Author contributions

NT: Writing—review & editing. TW: Writing—review & editing. JI: Writing—original draft, Writing—review & editing.
